# Phosphorus recovery from high-COD and strongly acidic wastewater by vivianite crystallization: feasibility and optimization of operating conditions

**DOI:** 10.3389/fchem.2026.1827333

**Published:** 2026-05-04

**Authors:** Kai Cui, Guangyu Xu, Fei Ma, Hong Zhang, Jiahao Cao, Yan Yi, Kun Guo

**Affiliations:** School of Chemical Engineering and Technology, Xi’an Jiaotong University, Xi’an, China

**Keywords:** acidified oil wastewater, optimization of operating conditions, phosphorous recovery, process prediction model, response surface methodology, vivianite crystallization

## Abstract

Acidified oil wastewater contains high concentrations of phosphorus and organic pollutants, which not only pose environmental risks but also contain recoverable phosphorus resources. However, its complex matrix (high COD and acidic environment) poses great challenges to phosphorus recovery technologies. This study successfully developed a new phosphorus recovery strategy adapted to the wastewater characteristics and optimized key operating parameters using response surface methodology. Key parameters including pH, Fe:P molar ratio, reaction time and residence time were investigated, clarifying their regulatory roles in the complex wastewater system. Under the optimal process conditions (pH = 7.77, Fe:P molar ratio = 2.24, reaction time = 2.23 h, and hydraulic residence time = 4.16 h), the phosphorus recovery rate reached 93.54%, and the vivianite crystallization rate reached 91.39%. Furthermore, a process parameter prediction model (the verification results and the model prediction was less than 2.1%) was established to produce vivianite crystallization with large particle size (d_50_ = 113.4 μm) and uniform morphology, realizing synchronous wastewater treatment and phosphorus recovery. This study confirmed the feasibility and controllability of phosphorus recovery through vivianite crystallization in complex acidified oil wastewater matrices, providing a sustainable technical path for addressing the dual issues of phosphorus resource shortage and environmental pollution.

## Graphical Abstract

**Figure:**
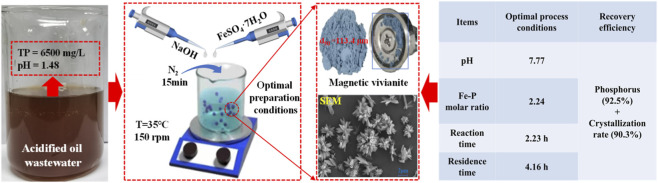

## 1 Introduction

Acidified oil, a crucial industrial intermediary derived from the sulfuric acid treatment of vegetable oil soap foot, is a cornerstone to synthesizing various chemicals such as oleic acid, fatty acids and biodiesel. With Chinese annual output reaching 800000 tons in 2020, the acidified oil underscores an important role in China manufacturing networks (Fan et al., 2023; Xu et al., 2019). However, the production process generates highly acidic wastewater streams exhibiting elevated oil content and significant phosphorus concentrations (Thant and Kim, 2021; Li et al., 2021). This acidified oil wastewater poses a formidable environmental threat, with potential repercussions for aquatic ecosystems and surrounding habitats (Xu et al., 2023).

As an indispensable non-renewable element, phosphorus is central to all living organisms and modern agricultural systems reliant on phosphate fertilizers (Li et al., 2020). However, global phosphorus reserves exhibit pronounced geographical asymmetry and limited availability, leading to potential food security concerns, market price volatility and geopolitical resource conflicts (Daneshgar et al., 2018). These challenges are intensified by the global population explosion and associated increases in food demand, which would increase phosphorus consumption and heighten the urgency for sustainable resource management strategies (Wu et al., 2016). In this context, recovering phosphorus from acidified oil wastewater emerges as an effective approach to mitigate water pollution while simultaneously addressing phosphorus scarcity concerns.

Over the past century, chemical precipitation has stood out as the most effective method for phosphorus recovery from industrial wastewater. The most common recovery techniques include struvite (Amann et al., 2018), hydroxyapatite (Petzet and CorneL, 2012), and vivianite precipitation (Wu et al., 2019). These methods involve adding metal ions such as magnesium, calcium, or iron to phosphorus-containing wastewater to form insoluble phosphate crystals that precipitate out, achieving the separation and recovery of phosphorus. However, when iron is added, iron ions preferentially react with phosphate to form vivianite (Fe_3_(PO_4_)_2_·8H_2_O), which differs structurally from struvite, preventing struvite recovery and limiting the method’s use in iron-containing systems (Wilfert et al., 2018). Currently, most vivianite studies focus on digest sludge or low-difficulty wastewaters (e.g., low chemical oxygen demand (COD), low pH, and simple composition) (Hao et al., 2022; Heinrich et al., 2023), with only one pilot-scale study achieving 80% recovery (Wijdeveld et al., 2022). Since vivianite is often formed *in situ* within the reactor, where uncontrolled nucleation and growth yield small, irregular crystals prone to co-precipitation with solids, complicating solid–liquid separation. Therefore, high-gradient magnetic separation is often required due to its weak magnetism, increasing process complexity and operational costs (Amin et al., 2024; Wijdeveld et al., 2022).

In contrast, vivianite precipitation is better suited for treating acidified oily wastewater. It forms under relatively low pH conditions (5.0–7.0) (Priambodo et al., 2017), whereas struvite and hydroxyapatite require pH above 8.5 and around 9.0, respectively, for stable precipitation (Hao et al., 2008). This allows vivianite to crystallize spontaneously in acidic conditions without extensive pH adjustment, reducing alkali use and operating costs. Moreover, vivianite exhibits good crystallinity, larger particle size potential, and higher market value due to its high iron content and unique crystal structure (Li et al., 2015). Therefore, exploring the efficacy of vivianite precipitation for phosphorus recovery from acidified oil wastewater holds great interest and significance (Chen et al., 2018).

Apart from high phosphate concentrations, acidified oil wastewater contains high levels of organic pollutants (reflected by high-COD, 40000–60000 mg/L), soluble salts (>6,000 mg/L), and complex refractory organics such as residual oil, fatty acids, and humic substances (Jiang et al., 2022). These components exert notable interference effects on vivianite crystallization. On one hand, abundant organic macromolecules and colloidal impurities tend to adsorb onto the surface of vivianite nuclei, hindering crystal nucleation and subsequent growth, reducing crystal regularity, and weakening the binding between phosphate and Fe^2+^, thus lowering phosphorus crystallization efficiency (Liu et al., 2026). On the other hand, high concentrations of Ca^2+^ and Mg^2+^ compete with Fe^2+^ for phosphate ions, generating amorphous calcium/magnesium phosphate precipitates and consuming excessive phosphate, which suppresses vivianite formation and reduces crystallization purity (Jiang et al., 2025). Additionally, high salinity elevates solution ionic strength, changing solution supersaturation and affecting the precipitation thermodynamics and kinetics of vivianite crystallization (Liu et al., 2018).

Based on the above background, this study aims to investigate the potential of recovering phosphate from such wastewater by utilizing vivianite crystallization. Firstly, it verifies the feasibility of recovering phosphorus from acidified oil wastewater through vivianite crystallization; secondly, it systematically optimizes the key operating parameters based on single-factor optimization experiments and response surface methodology (RSM). The study focuses on investigating the effects of pH value, Fe:P molar ratio, reaction time, and residence time on vivianite crystallization and phosphorus recovery efficiency, and clarifies the regulatory roles of each parameter in the complex wastewater system. Finally, a process parameter prediction model is established to obtain the optimal operating parameters for vivianite crystallization production, realizing synchronous wastewater treatment and phosphorus recovery. This study will confirm the feasibility and controllability of phosphorus recovery through vivianite crystallization in the complex matrix of acidified oil-containing wastewater, and provide a sustainable technical approach for solving the dual problems of phosphorus resource shortage and environmental pollution.

## 2 Materials and methods

### 2.1 Characteristics of acidified oil wastewater

The experimental raw wastewater was sourced from acidified vegetable oil wastewater collected from a fatty acid plant located in Weifang, Shandong Province. The main physicochemical characteristics of the wastewater are as follows: highly acidic with a pH of 1.48 ± 0.2, COD of 48000 ± 2000 mg/L, ammonium nitrogen (NH_4_^+^-N) concentration of 550 ± 50 mg/L, total phosphorus (TP) content of 6,500 ± 300 mg/L, Ca^2+^ concentration of 300 mg/L, and Mg^2+^ concentration of 500 mg/L. All water quality indicators were determined following standard Chinese examination methods for wastewater and sewage: COD was measured via the potassium dichromate digestion-spectrophotometry method (Kolb et al., 2017); NH_4_^+^-N was quantified by Nessler’s reagent spectrophotometry (Wu et al., 2013); Ca^2+^ and Mg^2+^ concentrations were analyzed using inductively coupled plasma-optical emission spectroscopy (ICP-OES) (Liu et al., 2020). The wastewater was stored in a refrigerator at 4 °C for subsequent experimental use.

### 2.2 Vivianite formation experiments

**FIGURE 1:**
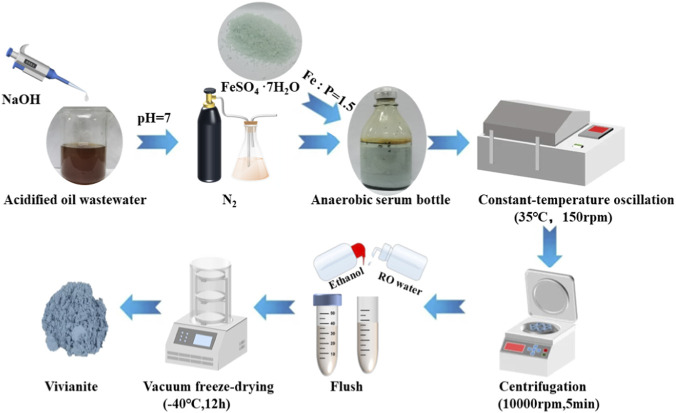
Flowchart of the vivianite crystallization process from acidified oil wastewater.

The process flow for the formation of vivianite crystals from acidified oil wastewater is illustrated in Figure 1. The detailed process steps are presented in Supplementary Figure S1. First, control the pH value of the wastewater sample to around 7 by adding 5M NaOH solution. Subsequently, high-purity nitrogen gas (purity ≥99.999%) was continuously bubbled into the acidified oil wastewater at a flow rate of 50 mL/min for 30 min to thoroughly remove dissolved oxygen and prevent the oxidation of Fe^2+^. The dissolved oxygen concentration in the solution was monitored in real time using a portable dissolved oxygen meter to ensure dissolved oxygen (DO) was reduced to below 0.2 mg/L, a level sufficiently low to avoid Fe^2+^ oxidation during the reaction. The Fe-P molar ratio in the solution was adjusted using FeSO_4_·7H_2_O. The solution was then placed in a thermostatic shaker incubator maintained at 35 °C with agitation at 150 rpm. After completion of the reaction, the suspension was centrifuged at 10000 rpm for 5 min, and the supernatant was collected for determination of residual total phosphorus concentration. The resulting precipitate was washed sequentially with reverse osmosis water and anhydrous ethanol, followed by vacuum freeze-drying at approximately −40 °C for 12.0 h to obtain the final product.

### 2.3 Analytical methods

The concentrations of PO_4_^3-^-P in the reaction solution were determined using the ascorbic acid method (Caban et al., 2025). TP was quantified by molybdenum-antimony inverse spectrophotometry, and Fe^2+^ concentrations were analyzed by inductively coupled plasma–optical emission spectroscopy (ICP-OES). In addition, the crystallographic characteristics of the precipitates, including morphology, elemental composition, and crystal structure, were evaluated by X-ray diffraction (XRD), scanning electron microscopy with energy-dispersive X-ray spectroscopy (SEM-EDX), and X-ray photoelectron spectroscopy (XPS). Particle size distribution was determined using an OMEC LS-909 laser diffraction analyzer, with the average particle size denoted as d_50_, which corresponds to the cumulative percentage of the particle size distribution at 50%. Further details regarding the analytical methods and instrument operational procedures can be found in a previous study (Amin et al., 2024; Prot et al., 2019).

The freeze-dried vivianite crystals were subjected to a stepwise extraction of phosphorus using the Hupfer method (Yang et al., 2023). During the Hupfer extraction process, phosphorus mainly exists in five forms: (1) Labile water-soluble phosphorus (Labile-P), which is easily dissolved in water and thus extracted using deionized water; (2) CO_3_^2-^ adsorbed phosphorus (MCO_3_-P), extracted using a 0.1 mol·L^-1^ acetic acid solution; (3) Iron (Fe)-bound phosphorus (Fe-P) with vivianite belonging to this category, aluminum (Al)-bound phosphorus (Al-P) and organic phosphorus (Org-P), extracted using 1 mol·L^-1^ NaOH; (4) Calcium-bound phosphorus (Ca-P), extracted using 0.5 mol·L^-1^ HCl; (5) Residual phosphorus (Residual-P), extracted through the digestion action of concentrated HNO_3_. Supplementary Table S1 presents the details of Phosphorus components in the Hupfer method. The phosphorus recovery (PR) efficiency (Equation 1) and crystallization rate (Equation 2) (namely, the ratio of crystallized phosphorus to total recovered phosphorus, CR) were calculated as follows:

$$\mathrm{PR}=\frac{{C_{pi}‐C}_{pe}}{C_{pi}}\times 100\%$$ (1)

$$CR=\frac{C_{Fe‐P}\times V\times Mv}{2\times Mp\times mr}\times 100\%$$ (2)

Where *C*_*pi*_ and *C*_*pe*_ represent the phosphorus concentration in the raw wastewater and the solution after the reaction, respectively; *Mp* denotes the molar mass of phosphorus, *Mv* is the molar mass of vivianite, *V* is the volume of extractant, *mr* is the mass of the freeze-dried vivianite crystal used for extraction, and *C*_*Fe-P*_ is the concentration of Fe-P determined via the Hupfer method.

### 2.4 Experimental design for the single-factor experiment and RSM

In this study, four variables (pH, Fe-P molar ratio, reaction time, and residence time) are selected to investigate their effects on the efficiency of PR and CR. RSM is a statistical method that uses a rational experimental design methods and empirical data to fit the functional relationship between factors and response values using a multiple quadratic regression equation. This method is utilized to identify optimal process parameters and address multivariate problems through the analysis of the regression equation (Jensen, 2017; Thakur et al., 2017; Yao et al., 2023).

In addition, different pH, Fe-P molar ratio, reaction time, and residence time were selected to evaluate the effects on PR and CR under single-factor experimental conditions. Subsequently, RSM was employed to analyze the combined effects of these variables on PR and CR and to model the conditions for maximizing response values. The analysis was conducted using Design-Expert 13 software, with PR and CR designated as the response variables. A Box-Behnken response surface experiment was conducted to analyze the influence of pH, Fe-P molar ratio, reaction time, and residence time on PR and CR in the treatment of acidified oil wastewater. In the experimental design, 27 groups with different experimental conditions were used to analyze the effect sizes of the four factors, and three replicate groups with the same experimental conditions were used to estimate the experimental error.

## 3 Result and discussion

### 3.1 Feasibility assessment of vivianite precipitation for phosphorus recovery

**FIGURE 2:**
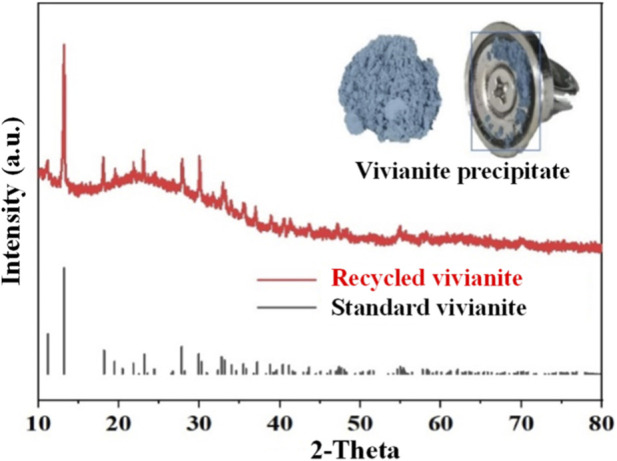
XRD patterns and morphological characteristics of the experimentally synthesized vivianite precipitates.

The feasibility of phosphorus recovery from acidified oil wastewater via the vivianite precipitation method was first verified. The low pH environment of acidified oil wastewater leads to phosphate mainly existing in the form of H_2_PO_4_^−^, and Fe^2+^ is prone to oxidation, which is unfavorable for the formation of vivianite. Therefore, sodium hydroxide was used to adjust the pH value to 7.0 (Figure 1). This pH condition not only effectively inhibits the oxidation of Fe^2+^ to provide a stable Fe^2+^ source for vivianite crystallization, but also promotes the conversion of H_2_PO_4_^−^ to HPO_4_^2-^, enhances the reactivity between phosphate and Fe^2+^, and creates a suitable thermodynamic environment for vivianite nucleation (Sl and omp Caroline, 2023). On this basis, the Fe-P molar ratio, reaction time, and residence time were set to 2:1, 2.0 h, and 4.0 h, respectively. Sufficient Fe^2+^ (Fe-P molar ratio of 2:1) can effectively overcome the adsorption and chelation of phosphate by complex organic matter in the wastewater, and simultaneously inhibit the competitive precipitation between coexisting ions (such as Ca^2+^ and Mg^2+^) and phosphate, ensuring the selectivity of vivianite crystallization. A reaction time of 2.0 h can meet the initial formation and growth of vivianite nuclei, while a residence time of 4.0 h can promote crystal ripening, reduce impurity adsorption on the crystal surface, and improve crystal purity. Finally, vivianite was successfully recovered through vivianite crystallization (Figure 2).

Figure 2 shows that the freeze-dried crystals are light blue powder and can be attracted by a magnet, which is due to the unpaired electrons of Fe^2+^ in the vivianite crystals having paramagnetism, making the crystals exhibit weak magnetism, further confirming the successful formation of vivianite (Matthias et al., 2016). The XRD pattern of the recycled vivianite is highly consistent with the standard pattern of vivianite, with no obvious shift in the position and intensity of characteristic diffraction peaks and no other impurity peaks, indicating that the crystals have good crystallinity and high purity, with no obvious impurity phases formed. These results clearly confirm the successful recovery of vivianite crystals from acidified oil wastewater from three aspects: crystal morphology, magnetic characteristics, and phase composition, and also verify the feasibility of applying the vivianite precipitation method in complex wastewater matrices.

To further improve the recovery performance of vivianite and solve the inhibitory effect of complex wastewater matrices (high COD, coexisting ions, and organic matter) on vivianite crystallization, four key variables (pH value, Fe/P molar ratio, reaction time, and residence time) were selected to evaluate their effects on phosphorus recovery efficiency and crystallization rate in acidified oil wastewater, providing theoretical and experimental basis for subsequent process optimization.

### 3.2 Optimization of the operational conditions for vivianite crystallization

#### 3.2.1 Effects of pH and Fe-P molar ratio on PR and CR

**FIGURE 3:**
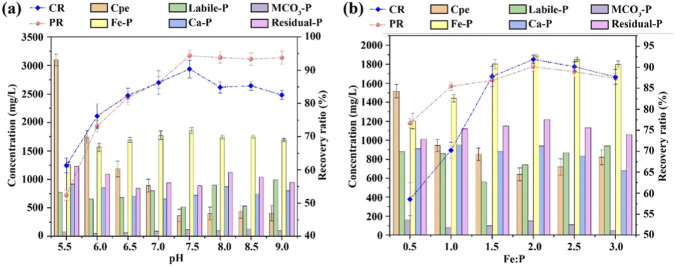
The variation characteristics of PR, CR and phosphorus distribution in recycled vivianite crystals under different pH values **(a)** and Fe-P molar ratios **(b)**.

Figure 3a displays the effects of pH value on PR and CR. As the pH value of the acidified oil wastewater increased from 5.5 to 7.5, both PR and CR increased significantly, reaching maximum values of 94.40% and 90.36%, respectively. However, when the pH value exceeded 7.5, PR remained stable, while CR decreased to 82.56%. In the acidified oil wastewater, elemental phosphorus mainly exists in the forms of H_2_PO_4_^−^) and HPO_4_^2-^). Due to the presence of interfering ions such as Ca^2+^ and Mg^2+^, the main reactions after adding ferrous sulfate to the solution are as follows:

$$3{\text{Fe}}^{2+}+2\text{HP}O_{4}^{2‐}+8H_{2}O\;\to {\;\text{Fe}}_{3}{\left({\text{PO}}_{4}\right)}_{2}\cdot 8H_{2}O+2H^{+}$$ (3)

$$3{\text{Fe}}^{2+}+2H_{2}PO_{4}^{2‐}+8H_{2}O\to {\;\text{Fe}}_{3}{\left({\text{PO}}_{4}\right)}_{2}\cdot 8H_{2}O+4H^{+}$$ (4)

$$3{\text{Ca}}^{2+}/{\text{Mg}}^{2+}+2\text{HP}O_{4}^{2‐}+8H_{2}O\;\to \text{ Ca}/{\text{Mg}}_{3}{\left(PO_{4}\right)}_{2}\cdot 8H_{2}O+2H^{+}$$ (5)

$$3Ca^{2+}/{\text{Mg}}^{2+}+2H_{2}PO_{4}^{2‐}+8H_{2}O\;\to \text{ Ca}/{\text{Mg}}_{3}{\left(PO_{4}\right)}_{2}\cdot 8H2O+4H^{+}$$ (6)

$${\text{Ca}}^{2+}/\;{\text{Mg}}^{2+}+2OH^{‐}\to \left(\text{Ca},\text{Mg}\right){\text{OH}}_{2}$$ (7)

Therefore, a certain amount of (Ca, Mg)_3_(PO_4_)_2_ and (Ca, Mg) (OH)_2_ precipitates may also be formed when the pH exceeds 7.5, which affects the yield of vivianite but does not impact the recovery efficiency of phosphorus. Consequently, a pH of 7.5 is determined to be optimal.

The theoretical Fe-P molar ratio for vivianite synthesis is 1.5:1.0 (Equations 3, 4). However, since acidified oil wastewater contains interfering ions such as Ca^2+^ and Mg^2+^ that compete with Fe^2+^ for phosphate (Equations 5, 6), an excess of Fe^2+^ is required in practice to ensure effective phosphorus recovery. Figure 3b shows the effects of different Fe-P molar ratios (0.5–3.0) on PR and CR. With the increase of Fe-P molar ratio, the efficiencies of PR and CR first increased and then decreased, which is the result of the synergistic effect of Fe^2+^ supply, interfering ion competition, and vivianite crystallization thermodynamics. When the Fe-P molar ratio increased from 0.5 to 2.0, PR increased from 76.67% to 90.15%, and CR increased from 58.58% to 91.88%. These phenomena are attributed to the insufficient supply of Fe^2+^ when the initial Fe-P molar ratio is 0.5:1.0, which not only fails to meet the stoichiometric requirements for vivianite synthesis but also cannot resist the competition of Ca^2+^ and Mg^2+^ for phosphate, resulting in a large amount of unreacted phosphate and low PR and CR. As the Fe-P molar ratio gradually increases, the supply of Fe^2+^ increases correspondingly. On one hand, Fe_2_^+^ can fully combine with phosphate in the wastewater, promoting the forward progress of the vivianite crystallization reaction. On the other hand, the excess Fe^2+^ can occupy the binding sites of phosphate, inhibit the competitive reaction of interfering ions, and reduce the formation of by-products, thereby significantly improving the PR and CR.

Although the Fe-P molar ratio is increased to 1.5:1.0, where the dosage of Fe^2+^ just meets the theoretical stoichiometric requirement for vivianite synthesis and achieves a good recovery rate, there are still some interfering ions remaining unregulated in system. A small amount of phosphate will combine with Ca^2+^ and Mg^2+^ in the wastewater to form non-target precipitates (Equation 7), resulting in incomplete conversion of phosphate into vivianite, and thus the phosphorus recovery efficiency cannot reach the optimal level. When the Fe-P molar ratio is 2.0, the appropriately excessive Fe^2+^ can effectively occupy the binding sites of phosphate, inhibit the competitive reaction between interfering ions and phosphate, and ensure that most of the phosphate in the system combines with Fe^2+^ to form stable vivianite precipitation. This not only improves the crystallization efficiency of vivianite but also avoids the loss of phosphate caused by the formation of non-target precipitates, thereby maximizing the phosphorus recovery rate.

When the Fe-P molar ratio exceeds 2.0, the excessive Fe^2+^ will lead to an abnormal increase in the concentration of Fe^2+^ in the system. On one hand, the excessive Fe^2+^ will undergo hydrolysis reaction in the aqueous solution, consuming a large amount of OH^−^ in the system, which further changes the pH of the solution and destroys the stable crystallization environment required for vivianite formation. On the other hand, the excessive Fe^2+^ will adsorb on the surface of the vivianite crystal, hindering the growth of the crystal and reducing the regularity of the crystal structure, which in turn affects the combination of Fe^2+^ and phosphate, leading to a decrease in phosphorus recovery rate and precipitation efficiency. Therefore, considering the phosphorus recovery effect, vivianite crystallization quality and reagent economy comprehensively, the optimal Fe-P molar ratio was determined to be 2.0.

#### 3.2.2 Effects of reaction time and residence time on PR and CR

**FIGURE 4:**
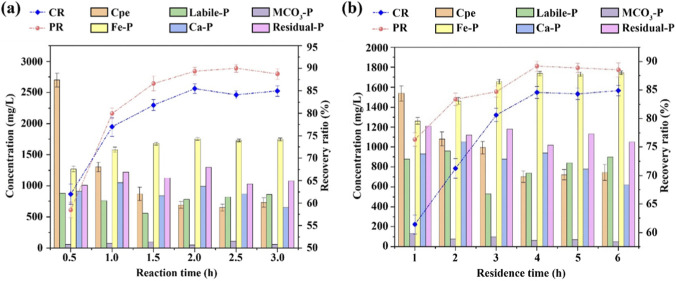
Changes in PR, CR and phosphorus distribution in recycled vivianite crystals under different reaction time **(a)** and residence time **(b)**.

Figure 4a shows that the yield and crystallization rate increased with the reaction time ranging from 0.5 h to 2.0 h. The yield reached the maximum value of 89.98% at 2.5 h, while the crystallization rate peaked at 85.47% at 2.0 h. This phenomenon may be attributed to the fact that the formation of vivianite is a dynamic kinetic process involving “nucleation-crystal growth”. A short reaction time (≤0.5 h) can only trigger the initial reaction between Fe^2+^ and phosphate, failing to provide sufficient kinetic time for the precipitation reaction. As a result, the yield and crystallization rate remain at a low level. With the reaction time extended to 2.0 h, Fe^2+^ undergoes a sufficient coordination reaction with H_2_PO_4_^−^ and HPO_4_^2-^ in the wastewater, leading to the massive formation and rapid growth of vivianite nuclei. The crystal structure is gradually improved and the crystallinity is continuously enhanced, thus the yield and crystallization rate increase steadily. When the reaction time exceeds 2.0 h, Fe^2+^ in the system has fully reacted with H_2_PO_4_^−^ or HPO_4_^2-^, and the phosphate has been basically completely converted into vivianite, leading the system to reach a state of reaction equilibrium. At this point, further extending the reaction time cannot promote the forward reaction, and the yield and crystallization rate remain relatively stable. Considering comprehensively the crystallization efficiency, reaction economy and operational convenience, the optimal reaction time was determined to be 2.0 h.

Figure 4b shows the effect of residence time on PR and CR. From 1.0 h to 4.0 h, both PR and CR increased with the extension of residence time. These results are mainly attributed to the fact that residence time directly affects the ripening process of vivianite crystals. The longer the residence time, the more conducive it is to the positive shift of the thermodynamic equilibrium of crystal growth (Liu et al., 2018). On one hand, sufficient residence time can reduce the agglomeration of vivianite crystals, allowing the nuclei enough time for oriented growth, thereby improving the crystal structure and crystallization purity. On the other hand, extending the residence time can promote the further combination of unreacted Fe^2+^ with residual phosphate in the system, reduce the loss of target products, and simultaneously inhibit the secondary reaction between interfering ions (such as Ca^2+^ and Mg^2+^) and phosphate, thus improving PR and CR synchronously. When the residence time reached 4.0 h, both PR and CR tended to be stable. The main reason is that the concentrations of Fe^2+^ and phosphate in the system have dropped to a low level, which can no longer maintain the solution supersaturation required for the continuous formation of vivianite crystals, and the crystal growth process has reached a dynamic equilibrium at this time. In summary, the optimal residence time was determined to be 4.0 h, which can achieve the optimal balance between phosphorus recovery efficiency and crystal quality.

### 3.3 Mathematical model for the optimal operating conditions of vivianite crystallization

Based on the results of single-factor preliminary optimization for wastewater treatment with the goal of phosphorus recovery, pH value, Fe:P molar ratio, reaction time, and hydraulic residence time were selected as key process decision variables. Among them, pH = 7.5, Fe:P = 2.0, reaction time = 2.0 h, and hydraulic residence time = 4.0 h were set as the central levels of each variable (coded value = 0). RSM was adopted to carry out multi-variable synergistic optimization (Yao et al., 2023): through systematic variable screening, orthogonal central composite design (CCD) experimental layout (Supplementary Table S2), quadratic polynomial response surface modeling, and regression significance test (including model F-test, lack-of-fit analysis, and evaluation of coefficient of determination *R* ^2^ and adjusted *R* ^2^), the optimal process parameter combination for the vivianite crystallization process was finally determined. The design results of Box-Behnken are shown in Table 1.

**TABLE 1:** The results of the Box-Behnken design.

A: pH	B: Fe-P	C: Reaction time (h)	D: Residence time (h)	PR	CR	Particle size (μm)
7.5	2	3	3	89.37	87.85	142.85
8.5	2	3	4	90.48	89.28	151.23
6.5	2	3	4	85.90	85.46	131.46
7.5	3	2	5	91.28	88.48	127.25
7.5	2	3	5	89.23	87.23	147.23
8.5	2	2	3	88.08	85.72	131.72
6.5	3	2	4	84.70	83.48	123.49
7.5	2	2	4	92.76	91.36	164.77
7.5	2	2	4	93.36	90.85	166.85
7.5	3	2	3	87.83	84.25	138.25
6.5	2	1	4	85.66	85.82	127.83
7.5	1	3	4	86.75	83.86	125.86
8.5	2	1	4	86.25	85.83	145.83
7.5	1	1	4	87.15	83.82	117.82
6.5	2	2	3	85.32	85.52	137.53
7.5	1	2	5	87.46	83.90	125.91
7.5	3	3	4	89.83	88.95	144.95
7.5	2	1	3	89.21	87.21	140.22
7.5	1	2	3	86.52	86.46	121.47
8.5	1	2	4	84.78	83.75	119.75
8.5	3	2	4	89.89	87.89	145.89
7.5	3	1	4	87.72	86.08	138.08
6.5	2	2	5	85.86	84.75	130.75
6.5	1	2	4	83.92	84.21	118.21
8.5	2	2	5	90.95	87.83	147.84
7.5	2	1	5	88.85	87.37	138.37
7.5	2	2	4	92.85	90.76	163.36

#### 3.3.1 Response surface analysis for PR

**FIGURE 5:**
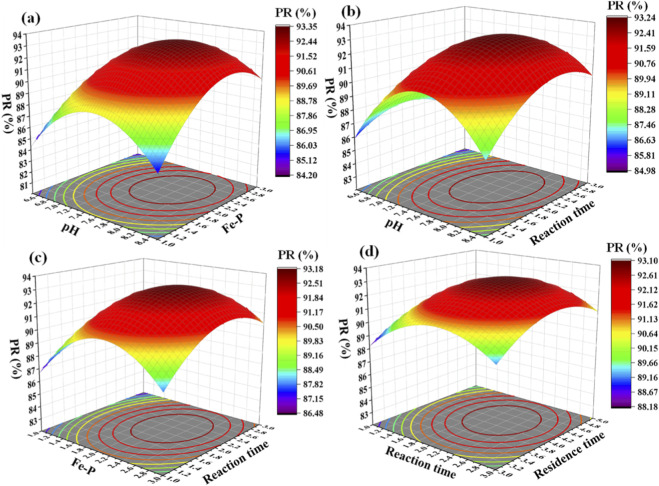
The 3D response surface plot with PR as the response index. pH and Fe-P **(a)**, pH and reaction time **(b)**, Fe-P and reaction time **(c)**, and reaction time and residence time **(d)**.

The 3D response surface plot (Figure 5) with PR as the response index and the response surface variance analysis (Supplementary Table S3) indicate that this model can reliably describe the quantitative relationship between various process parameters and PR, and thus can be used for the prediction of PR and process optimization. Among them, the F value of the model is 19.91 (far higher than the critical value), which indicates that the regression model is statistically significant. From the perspective of the 3D response surface plots, the terms A (pH), B (Fe-P molar ratio), C (reaction time), D (residence time), interaction terms such as AB and AC, and quadratic terms such as A^2^ and B^2^ in the model are all significant model terms (all p-values <0.05), which is closely related to the inherent law of vivianite crystallization. The pH value affects the nucleation efficiency of crystal nuclei by regulating the oxidative stability of Fe^2+^ and the speciation of phosphate (conversion between H_2_PO_4_^−^ and HPO_4_^2-^). The Fe:P molar ratio influences the crystallization selectivity by adjusting the ratio of Fe^2+^ to phosphate and the competitive effect of interfering ions. The reaction time and residence time affect the nucleation kinetics and crystal ripening process, respectively. The significance of the interaction terms indicates that each parameter does not act independently but has synergistic or antagonistic effects (for example, the interaction between pH value and Fe:P molar ratio can jointly regulate the reactivity of Fe^2+^ with phosphate, thereby affecting PR). The significance of the quadratic terms indicates that the influence of each parameter on PR is not a linear relationship but has an optimal range. Beyond this range, PR will decrease due to system imbalance (such as Fe^2+^ hydrolysis caused by excessively high pH and crystal agglomeration caused by excessively high Fe:P molar ratio).

In addition, the F-value of the lack-of-fit test is 6.93 and the P-value is 0.1326 (>0.05), indicating that there is no significant lack-of-fit in the model. This demonstrates that the quadratic regression model can fully fit the experimental data without missing key influencing factors, and can accurately reflect the inherent correlation between process parameters and PR. The difference between the predicted *R* ^2^ (0.7663) and the adjusted *R* ^2^ (0.9106) is less than 0.2, indicating that the model has a good fit, with a small deviation between the predicted values and the actual experimental values, which further confirms the reliability of the model. The coefficient of variation (C.V. = 1.43% < 10%) indicates that the experimental data have excellent repeatability and small experimental errors; the model can explain 91.06% of the changes in PR, and the fitting accuracy meets the optimization requirements. Meanwhile, the signal-to-noise ratio is 14.979, which is much higher than the recommended minimum value of 4.0, indicating that the model has sufficient discriminant ability and can effectively distinguish the effects of different process parameter combinations on PR. Based on the above statistical indicators, it is confirmed that the model can be used for the prediction of PR and the optimization of experimental conditions in the vivianite crystallization process.

According to the magnitude of the F-value, the order of the influence of the four factors on PR is: pH value > Fe:P molar ratio > residence time > reaction time. This order is essentially consistent with the influence weight of each factor on the vivianite crystallization process. As the primary influencing factor, the pH value directly determines the stability of Fe^2+^ and the reactivity of phosphate, which is a prerequisite for the nucleation of vivianite crystal nuclei. The Fe:P molar ratio is the second most important factor; by regulating the excess degree of Fe^2+^, it inhibits the competition of interfering ions and determines the conversion efficiency of phosphate. The residence time and reaction time mainly affect the crystal ripening and growth processes, and have a relatively weak influence on PR. The multiple quadratic response surface regression model for PR obtained by regression analysis is: PR = 92.99 + 1.59A+ 1.22B+ 0.5601C + 0.6083D + 1.08AB+ 0.9975C + 0.5825AD + 0.6275BC + 0.6275BD + 0.055CD - 3.90A^2^ - 3.14B^2^ - 2.07C^2^ - 1.62D^2^. This model quantifies the influence degree of each parameter and their interactions on PR, providing a theoretical basis for the solution of the optimal process parameters in the subsequent steps.

#### 3.3.2 Response surface analysis for CR

**FIGURE 6:**
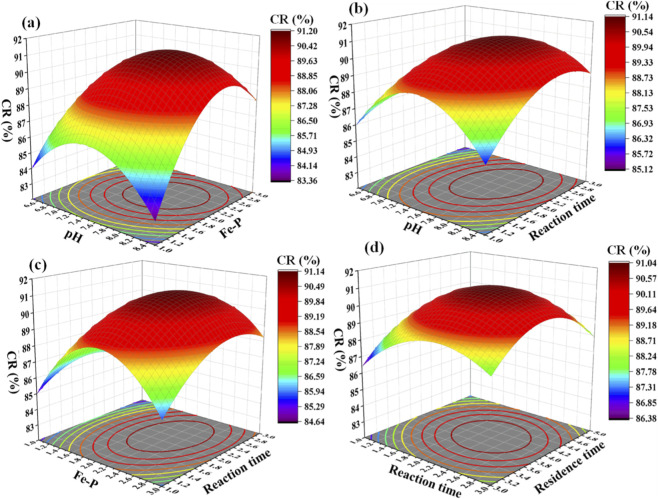
The 3D response surface plot with CR as the response index. pH and Fe-P **(a)**, pH and reaction time **(b)**, Fe-P and reaction time **(c)**, and reaction time and residence time **(d)**.

In this study, PR focuses on the degree of phosphorus recovery, while CR emphasizes the crystallization efficiency and purity of vivianite crystals, with subtle differences in their influence mechanisms. The 3D response surface plot (Figure 6) and the ANOVA results of the response surface model (using the ratio of crystallized phosphorus to total recovered phosphorus (CR) as the response variable) are presented in Supplementary Table S4. The F-value of the model is 18.97, indicating that the model has a good fitting effect and can reliably describe the relationship between various process parameters and CR. From the perspective of significance analysis, terms A, B, C, interaction terms such as AB and AC, and quadratic terms such as A^2^ and B^2^ are significant model terms (p-value <0.05). This phenomenon is mainly because the Fe:P molar ratio, as the primary influencing factor of CR (different from the influence order of PR), directly determines the nucleation quantity and purity of vivianite crystals. Excess Fe^2+^ can inhibit the formation of secondary precipitates and improve crystallization purity. The pH value affects the perfection of the crystal structure by regulating the crystal growth environment. The reaction time influences the sufficiency of crystal nucleus growth, thereby affecting the crystallization rate. The significance of interaction terms (such as AB and AC) indicates that the synergistic effects between parameters jointly regulate the crystal growth process and affect the crystallization effect.

In addition, the F-value of the lack-of-fit test for the CR model is 5.64 and the P-value is 0.1599 (>0.05), confirming that the model fits the experimental data well without significant lack-of-fit. The difference between the predicted *R* ^2^ (0.7561) and the adjusted *R* ^2^ (0.9063) is less than 0.2, and the coefficient of variation of the model is 0.8207% (C.V. = 0.8207% < 10%). The signal-to-noise ratio is 14.9909 (much higher than 4.0). All statistical indicators meet the requirements for model reliability, confirming that the model can be used for the prediction of CR and the optimization of experimental conditions. According to the magnitude of the F-value, the order of the influence of the four factors on CR is: Fe:P molar ratio > pH value > reaction time > residence time. This order reflects the core influencing factors of crystal purity and crystallization efficiency, that is, the Fe:P molar ratio directly determines the selectivity of vivianite crystallization and is the key factor affecting CR. The pH value regulates the crystal growth environment and has the second most significant influence on the crystallization rate. The reaction time affects the sufficiency of crystal nucleus growth, while the residence time has a relatively weak influence on crystal ripening. The quadratic polynomial regression equation for CR obtained from the model is as follows: CR = 90.99 + 0.9217A+ 1.09B+ 0.5417C + 0.2125D + 1.22AB+ 0.9525AC + 0.72AD + 0.7075BC + 1.70BD - 0.195CD - 2.84A^2^ - 3.39B^2^ - 1.69C^2^ - 1.97D^2^. This equation quantifies the influence law of each parameter on CR, complements the PR regression model, and provides quantitative support for the comprehensive optimization of the vivianite crystallization process.

#### 3.3.3 Response surface analysis for particle size

**FIGURE 7:**
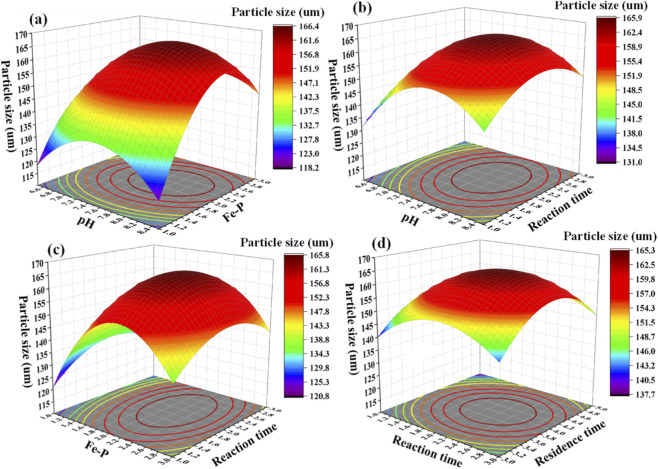
The 3D response surface plot with particle size as the response index. pH and Fe-P **(a)**, pH and reaction time **(b)**, Fe-P and reaction time **(c)**, and reaction time and residence time **(d)**.

Figure 7 and Supplementary Table S5 show that the model F-value is 21.62 with a model P-value less than 0.05, indicating that the model terms are statistically significant and can reliably describe the relationship between process parameters and vivianite particle size (d_50_). In this case, terms A, B, C, interaction terms AB and AD, and quadratic terms A^2^, B^2^, C^2^, and D^2^ (D = standing time) are important model terms (p < 0.1), which are closely related to the crystallization behavior of vivianite and further affect its separation potential. The signal-to-noise ratio is 16.135 (>4), indicating sufficient signal strength to accurately analyze the total phosphorus removal effect, which is essentially associated with the crystallization completeness of vivianite and the subsequent solid-liquid separation efficiency.

Meanwhile, the lack-of-fit P-value is 0.1512 (>0.05) and the lack-of-fit F-value is 6.00, meaning that the lack-of-fit is not significant relative to the pure error. This is favorable for the model, and there is no factor causing loss of fit, ensuring that the model can accurately reflect the influence of process parameters on vivianite crystallization behavior (e.g., crystal growth and agglomeration) and particle size distribution. The predicted *R* ^2^ is 0.7847, which is consistent with the adjusted *R* ^2^ of 0.9174 (>0.80) with a difference of less than 0.2, indicating that the regression model can fully explain the process of vivianite particle size formation, including the intrinsic link between process optimization, crystallization behavior and separation potential. The coefficient of variation (C.V.% = 2.90% < 10%) indicates excellent repeatability of experimental data, and only 7.10% of the variance cannot be explained by the model, further confirming the reliability of the model in guiding the optimization of vivianite crystallization and separation processes. The regression equation is: d_50_ = 164.99 + 6.09A+ 7.41B+ 2.96C + 0.4427D + 5.22AB+ 0.4553AC + 5.72AD - 0.2935BC - 3.86BD + 1.56CD - 15.21A^2^ - 23.29B^2^ - 10.18C^2^ - 12.98D^2^. As indicated by the F-values, the order of the influence degree of each factor on the vivianite particle size is: Fe:P molar ratio > pH value > reaction time > standing time. This order directly reflects the key role of each parameter in regulating vivianite crystallization behavior: the Fe:P molar ratio, as the most influential factor, determines the nucleation quantity and crystal growth rate of vivianite, thereby affecting particle size distribution and separation potential; pH value regulates the crystal growth environment and reduces crystal agglomeration, which is conducive to forming uniform particle sizes and improving solid-liquid separation efficiency; reaction time and standing time mainly affect the sufficiency of crystal growth and ripening, and their appropriate regulation can further optimize particle size characteristics and enhance the separation performance of vivianite from wastewater.

#### 3.3.4 Model prediction of the optimal combination of process parameters

**FIGURE 8:**
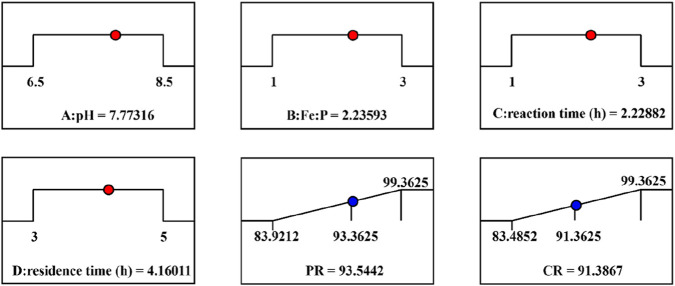
The optimal process conditions for the crystallization of vivianite based on model prediction.

Based on the quadratic polynomial prediction model constructed by RSM, multi-objective synergistic optimization was carried out for the vivianite crystallization process in the acidified oil wastewater system (Figure 8). The optimal combination of process parameters was determined as follows: pH = 7.77, Fe:P molar ratio = 2.24, reaction time = 2.23 h, and hydraulic residence time = 4.16 h. Under these conditions, the PR reached 93.54%, the vivianite CR reached 91.39%, and the comprehensive optimization index (desirability index) reached 0.568. The deviation between the verification results and the model prediction was less than 2.1%, indicating that the model has good prediction accuracy and engineering applicability.

### 3.4 Characteristics of vivianite crystallization under the optimal conditions

**FIGURE 9:**
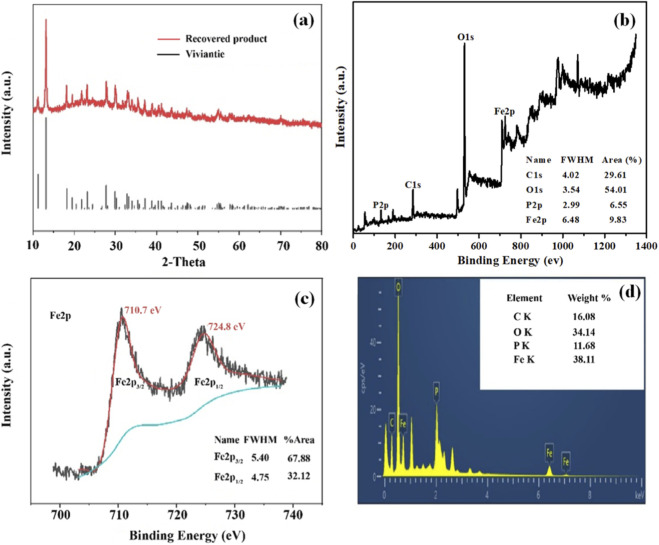
The phase structure characteristics of vivianite crystallization under the optimal conditions. XRD pattern **(a)**, XPS spectra **(b,c)**, and SEM-EDS **(d)** profiles of the precipitation.

Figure 9 illustrates the phase structure characteristics of the recovered product under the optimal process operating conditions, which is closely related to the crystallization behavior optimized by the RSM model and directly determines the separation potential and product purity of vivianite. The XRD pattern of the recovered vivianite product (Figure 9a) matches well with the standard vivianite diffraction pattern at 2θ angles of 11.188°, 13.164°, and 18.144°, with no other or unknown peaks observed. This confirms that vivianite is the main crystalline phase in the recovered product, indicating that the optimized process parameters can effectively promote the selective crystallization of vivianite and inhibit the formation of impurity phases, which lays a foundation for the efficient separation and recovery of vivianite. The atomic composition and chemical state of iron in the recovered vivianite product were further investigated by XPS analysis (Figure 9b). The product exhibits distinct XPS peaks at 713.36, 530.3, 286.45, and 132.3 eV, corresponding to Fe2p, O1s, C1s, and P2p, respectively. The area ratio of Fe2p to P2p is 3:2, which is consistent with the stoichiometric ratio of the vivianite chemical formula (Lu et al., 2021), further verifying the successful formation of vivianite crystals under the optimized process conditions.

In Figure 9c, the binding energies at 710.7 eV and 724.8 eV correspond to Fe2p_3_/_2_ and Fe2p_1_/_2_ of Fe^2+^, respectively. In addition, the energy interval of 14.1 eV also confirms the presence of divalent iron in the crystalline state (Jensen, 2017). The EDS image (Figure 9d) also shows the uniform distribution and high content of the four constituent elements (C, Fe, P, and O) in the crystal, which indicates that the optimized process can promote the uniform growth of vivianite crystals and avoid the uneven distribution of elements caused by incomplete crystallization. The elemental composition of Fe and P on the crystal surface (Fe: 38.11%, P: 11.68%) is very similar to the theoretical values of vivianite (Fe: 34.58%, P: 12.76%), and the slight difference is mainly due to the adsorption of trace organic matter or impurities on the crystal surface, which does not affect the main phase composition and separation potential of vivianite.

**FIGURE 10:**
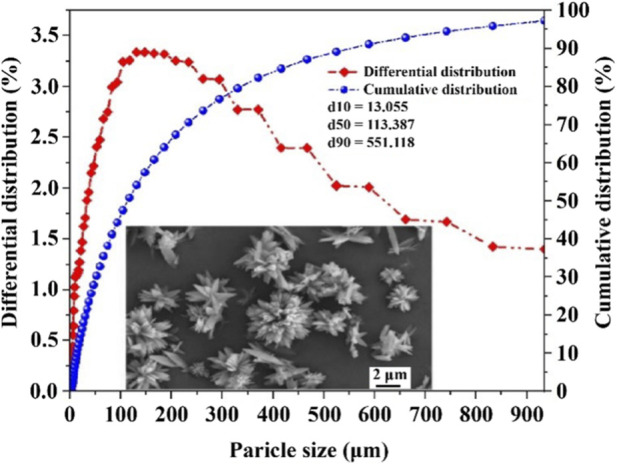
The particle size variation and morphological characteristics of vivianite crystallization formed under the optimal conditions.

Figure 10 shows that the crystal particle size (d_50_) achieved under optimal operating conditions was 113.387 μm, falling within the particle size range suitable for industrial applications (d_50_ = 80–120 μm) (Zhang et al., 2023), indicating the crystallization process under this design of process parameters is well controllable. Larger crystal sizes typically indicate complete crystal growth and a low nucleation rate, resulting from the effective control of supersaturation and optimized residence time in the reaction system, which promotes the formation of well-developed and structurally stable crystals. To assess measurement reliability, three parallel experiments were performed, yielding a relative standard deviation of 3.9%, below the acceptable threshold of 5%. This result demonstrates high repeatability and process stability, with minimal batch-to-batch variation, fulfilling the requirements for product consistency in large-scale production. Furthermore, microscopic morphological analysis of the recovered products was performed using SEM, revealing that the crystals exhibit typical flower-like and plate-like aggregates, with individual structural units measuring approximately 2.0 μm. The overall arrangement is orderly, the surfaces are smooth, and the boundaries are well-defined. This morphological is highly consistent with the literature-reported structure of vivianite (Guan et al., 2021), further supporting the preliminary phase identification of the product.

### 3.5 Technical feasibility analysis

Preliminary economic analysis indicates that this process holds favorable industrial application potential. The main operating costs include chemical reagents (FeSO_4_·7H_2_O and NaOH) and energy consumption (stirring, pH adjustment, and vacuum freeze-drying). Ferrous sulfate heptahydrate and sodium hydroxide are bulk industrial chemicals with low unit prices. The optimal Fe-P molar ratio (2.24) is moderate and does not introduce excessive reagent costs. Vacuum freeze-drying is only used for sample preparation in laboratory research, and can be replaced by low-cost mechanical centrifugation and hot air drying in industrial-scale production, significantly reducing energy consumption. Moreover, the recovered vivianite product is a high-value slow-release phosphate fertilizer and industrial raw material, whose economic benefits can offset part of the treatment costs. Combined with the high phosphorus recovery and crystallization efficiency, this technology realizes synchronous wastewater treatment and resource recovery, showing good economic and environmental benefits. Further cost optimization and scaling-up tests will be carried out in future research to promote industrial application.

The large-sized vivianite crystals obtained in this study offer multiple advantages for practical engineering applications. On one hand, larger particles exhibit a higher settling velocity during sedimentation, significantly enhancing solid–liquid separation efficiency and reducing energy consumption and equipment loading associated with subsequent filtration or centrifugation processes. On the other hand, due to their lower specific surface area, they adsorb fewer impurity ions on the surface, which contributes to higher final product purity and reduced washing requirements. Moreover, this characteristic effectively minimizes the loss of fine particles passing through filter media during separation, thereby improving the overall phosphorus recovery rate. Therefore, achieving large particle size and high uniformity in crystal growth is not only a key objective in crystallization process optimization but also provides robust technical support for the efficient recovery and potential commercialization of phosphorus resources.

## 4 Conclusion

This study confirms the feasibility of recovering phosphorus from acidified oil wastewater through vivianite crystallization. RSM-based optimization identified the optimal operational parameters (pH = 7.77, Fe:P molar ratio = 2.24, reaction time = 2.23 h, and hydraulic residence time = 4.16 h), which effectively mitigate the inhibitory effects of high COD, coexisting ions, and organic pollutants on vivianite formation. Under optimal conditions, high PR (93.54%) and CR (91.39%) are achieved, with the product being high-purity vivianite characterized by typical crystal morphology, stable phase composition, and suitable particle size for industrial separation. The process features low reagent costs, replaceable energy-consuming steps, and high-value products, offering favorable economic and environmental benefits. Large-sized vivianite crystals further enhance solid-liquid separation efficiency, supporting industrial scalability. This technology provides a promising solution for phosphorus resource recovery and acidified oil wastewater treatment.

## Data Availability

The original contributions presented in the study are included in the article/Supplementary Material, further inquiries can be directed to the corresponding author.
