# Supplementary material for: Phosphorus recovery from high-COD and strongly acidic wastewater by vivianite crystallization: feasibility and optimization of operating conditions
Source: Front Chem. 2026 May 4;14:1827333. doi: 10.3389/fchem.2026.1827333 (PMC13182519; doi:10.3389/fchem.2026.1827333)
Supplement: Supplementary file 1 [file DataSheet1.docx]

**Supporting Information**

**Phosphorus recovery from high-COD and strongly acidic wastewater by vivianite crystallization：Feasibility and optimization of operating conditions**

Kai Cui ^a^, Guangyu Xu ^a^, Fei Ma ^a^, Hong Zhang ^a^, Jiahao Cao ^a^, Yan Yi ^a^, Kun Guo ^a^*

^a^ *School of Chemical Engineering and Technology, Xi’an Jiaotong University, Xi’an 710049, China*

*Corresponding author: [*kun.guo@xjtu.edu.cn*](mailto:kun.guo@xjtu.edu.cn).


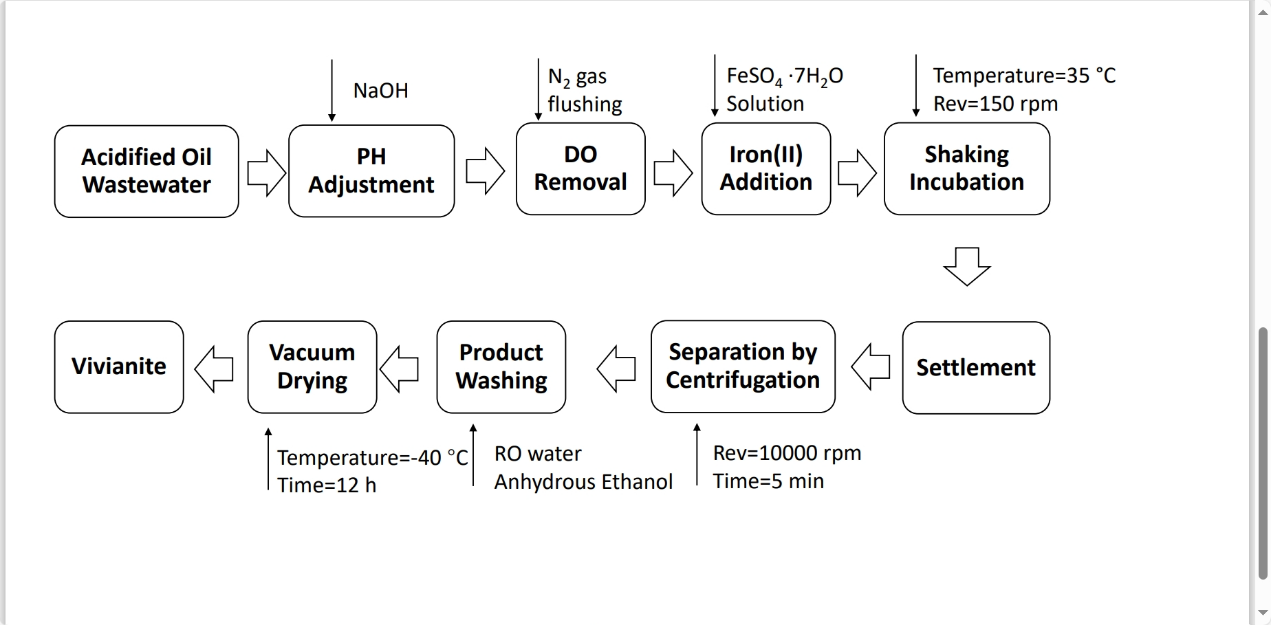


**Figure S1.** Flow chart of the vivianite crystallization from acidified oil wastewater.

**Table S1.** The Hupfer extraction method.

| Phosphorus  components | Extractant | Concentration  (mol/L) | Extraction duration (min) | Solid-to-liquid Ration  (mL: gDS) |
| --- | --- | --- | --- | --- |
| Labile-P  MCO_3_-P  (Fe-P+Al-P+Org-P)^a^  Ca-P  Residual-P | deionized water  acetic acid  NaOH  HCl  HNO_3_ | -  0.10  1.00  0.50  14.4 | 20  80  1080  1080  60 | 60:1  60:1  60:1  60:1  60:1 |

^a^ The total is regarded as Fe-P because the raw wastewater does not contain Al^3+^ or organic matter.

**Table S2.** The factor level of the Box-Behnken design.

| Factor | Level | | |
| --- | --- | --- | --- |
|  | -1 | 1 | 0 |
| pH | 6.5 | 7.5 | 8.5 |
| Fe/P | 1 | 2 | 3 |
| Reaction time (h) | 1 | 2 | 3 |
| Residence time (h) | 3 | 4 | 5 |

**Table S3.** The variance of response surface experiment based on PR.

| Source | Sum of squares | F-value | P-value |  |
| --- | --- | --- | --- | --- |
| Model | 171.86 | 19.91 | < 0.0001 | Significant |
| A | 30.32 | 49.17 | < 0.0001 |  |
| B | 17.95 | 29.11 | 0.0002 |  |
| C | 3.76 | 6.11 | 0.0294 |  |
| D | 4.45 | 7.22 | 0.0198 |  |
| AB | 4.68 | 7.59 | 0.0175 |  |
| AC | 4.00 | 6.49 | 0.0256 |  |
| AD | 1.35 | 2.19 | 0.1644 |  |
| BC | 1.58 | 2.56 | 0.1357 |  |
| BD | 1.59 | 2.58 | 0.1342 |  |
| CD | 0.01 | 0.02 | 0.8861 |  |
| A^2^ | 80.91 | 131.23 | < 0.0001 |  |
| B^2^ | 52.61 | 85.33 | < 0.0001 |  |
| C^2^ | 22.83 | 37.02 | < 0.0001 |  |
| D^2^ | 14.09 | 22.85 | 0.0004 |  |
| Lack of fit | 7.19 | 6.93 | 0.1326 | Not Significant |

Note: A denotes pH, B denotes Fe-P molar ratio, C denotes reaction time, and D denotes residence time.

**Table S4.** The variance of response surface experiment based on CR.

| Source | Sum of squares | F-value | P-value |  |
| --- | --- | --- | --- | --- |
| Model | 134.13 | 18.97 | < 0.0001 | Significant |
| A | 10.21 | 20.21 | 0.0007 |  |
| B | 14.37 | 28.44 | 0.0002 |  |
| C | 3.53 | 6.99 | 0.0214 |  |
| D | 0.54 | 1.07 | 0.3210 |  |
| AB | 5.92 | 11.72 | 0.0050 |  |
| AC | 3.65 | 7.23 | 0.0197 |  |
| AD | 2.08 | 4.12 | 0.0652 |  |
| BC | 2.00 | 3.95 | 0.0701 |  |
| BD | 11.56 | 22.89 | 0.0004 |  |
| CD | 0.15 | 0.29 | 0.5979 |  |
| A^2^ | 43.14 | 85.40 | < 0.0001 |  |
| B^2^ | 61.46 | 121.67 | < 0.0001 |  |
| C^2^ | 15.28 | 30.24 | 0.0001 |  |
| D^2^ | 20.58 | 40.75 | < 0.0001 |  |
| Lack of fit | 5.85 | 5.64 | 0.1599 | Not Significant |

Note: A denotes pH, B denotes Fe-P molar ratio, C denotes reaction time, and D denotes residence time.

**Table S5.** The variance of response surface experiment based on particle size.

| Source | Sum of squares | F-value | P-value |  |
| --- | --- | --- | --- | --- |
| Model | 4818.79 | 21.62 | < 0.0001 | Significant |
| A | 444.91 | 27.95 | 0.0002 |  |
| B | 658.50 | 41.36 | < 0.0001 |  |
| C | 105.07 | 6.60 | 0.0246 |  |
| D | 2.35 | 0.15 | 0.7075 |  |
| AB | 108.86 | 6.84 | 0.0226 |  |
| AC | 0.83 | 0.05 | 0.8233 |  |
| AD | 130.94 | 8.23 | 0.0141 |  |
| BC | 0.345 | 0.02 | 0.8855 |  |
| BD | 59.57 | 3.74 | 0.0770 |  |
| CD | 9.70 | 0.61 | 0.4501 |  |
| A^2^ | 1233.68 | 77.50 | < 0.0001 |  |
| B^2^ | 2892.74 | 181.71 | < 0.0001 |  |
| C^2^ | 553.00 | 34.74 | < 0.0001 |  |
| D^2^ | 899.16 | 56.48 | < 0.0001 |  |
| Lack of fit | 191.03 | 6.00 | 0.1512 | Not Significant |

Note: A denotes pH, B denotes Fe-P molar ratio, C denotes reaction time, and D denotes residence time.
